# Altered Orientation and Flight Paths of Pigeons Reared on Gravity Anomalies: A GPS Tracking Study

**DOI:** 10.1371/journal.pone.0077102

**Published:** 2013-10-23

**Authors:** Nicole Blaser, Sergei I. Guskov, Virginia Meskenaite, Valerii A. Kanevskyi, Hans-Peter Lipp

**Affiliations:** 1 Institute of Anatomy, University of Zurich, Zurich, Switzerland; 2 High-Technologies Institute, Kiev, Ukraine; 3 Ukrainian Geological Institute, Kiev, Ukraine; Université de Bordeaux and Centre National de la Recherche Scientifique, France

## Abstract

The mechanisms of pigeon homing are still not understood, in particular how they determine their position at unfamiliar locations. The “gravity vector” theory holds that pigeons memorize the gravity vector at their home loft and deduct home direction and distance from the angular difference between memorized and actual gravity vector. However, the gravity vector is tilted by different densities in the earth crust leading to gravity anomalies. We predicted that pigeons reared on different gravity anomalies would show different initial orientation and also show changes in their flight path when crossing a gravity anomaly. We reared one group of pigeons in a strong gravity anomaly with a north-to-south gravity gradient, and the other group of pigeons in a normal area but on a spot with a strong local anomaly with a west-to-east gravity gradient. After training over shorter distances, pigeons were released from a gravitationally and geomagnetically normal site 50 km north in the same direction for both home lofts. As expected by the theory, the two groups of pigeons showed divergent initial orientation. In addition, some of the GPS-tracked pigeons also showed changes in their flight paths when crossing gravity anomalies. We conclude that even small local gravity anomalies at the birth place of pigeons may have the potential to bias the map sense of pigeons, while reactivity to gravity gradients during flight was variable and appeared to depend on individual navigational strategies and frequency of position updates.

## Introduction

The mechanisms of long-distance orientation of birds are only partially understood. According to the map-and-compass theory by Kramer, the orientation process consists of two different parts [1]: a position-finding mechanism, and different mechanisms to determine and maintain directions. The latter include solar [2], stellar [3]–[4] and magnetic cues [5]–[8], landscape features [9] and polarized light patterns [10]–[11].

The position-finding mechanism, the map sense, is still unclear. Until now, two not mutually exclusive types of maps have been proposed: mosaic maps and gradient maps [12]–[14]. A mosaic map consists of experienced cues in a spatial frame, and is therefore mostly restricted to a familiar area (also called a familiar topographical map). Potential cues for a mosaic map are distinct visual landmarks [15]–[21] and airborne odors [22]–[23]; the latter also providing information about distant locations. On the other hand, a bi- or multicoordinate gradient map should have stable gradients and therefore should be extendable to unfamiliar areas. Potential candidates for a large-scale gradient map are olfactory cues, parameters of the earth's magnetic field and infrasound [24].

An olfactory gradient map is thought to consist of a global grid formed by intersecting relative proportions of volatile compounds allowing for homing and navigation by minimizing the difference of locally perceived versus remembered values of concentration at the home loft. A strong argument in favor of the olfactory hypothesis is that olfactory deprivation strongly interfered with homing and navigation [25]–[28]. One counter-argument is that the effect of olfactory deprivation was not related to navigation and that olfaction plays a role in activating the bird's navigational system [14], [29], a conclusion challenged by Gagliardo and colleagues [30] on the basis of GPS tracking.

The earth's magnetic field has also been considered as a candidate for a bicoordinate map formed by inclination angle and intensities of the geomagnetic field [13]–[14]. Releasing pigeons at local magnetic anomalies have shown some effects but the results and interpretations differ in these studies as homing itself is not severely affected [31]–[34]. Magnetic cues are subject to strong temporal and geographic variations. This casts some doubts whether they form the evolutionary backbone of a global positioning system for long-distance navigators [35]–[36].

Taken together, there is agreement that the navigational system of pigeons reflects the interaction of several mechanisms maintaining directions, but there is large disagreement about the mechanisms underlying the map sense. At least at present, it would seem that none of the proposed olfactory and magnetic mechanisms has the necessary robustness to account for the precision of avian long-distance navigation.Surprisingly, gravity itself has barely been considered as a possible cue for the orientation process. Larkin and Keeton [37] have found a significant correlation between the pigeons' mean vanishing bearings and the day of the lunar synodic month, suggesting that subtle changes in gravitational forces may influence navigation. Dornfeldt [38] conducted a thorough multivariate analysis of pigeon homing in relation to geomagnetic, gravitational, topographical and meteorological cues. He concluded that the most important factor accounting for poor homing orientation and performance was gravity anomalies. Kanevskyi and colleagues [39] followed pigeons by helicopter flying over a massive tectonic break (associated with a gravity anomaly). The pigeons altered their flight paths when crossing the anomaly and also showed some telemetrically assessed changes of the EEG. Conceptually related to the gravity vector theory, Köhler [40] proposed a navigation mechanism by assuming that the pigeons were able to use the visual horizon line for perceiving the difference between the horizontal plane at the home loft and the release site. On the other hand, Lednor and Walcott [41] released homing pigeons within weak negative gravitational anomalies (salt domes) but could not find a correlation with the homing orientation.

One theory explaining the possible use of gravity parameters for navigation is the “gravity vector” hypothesis proposed first by Kanevskyi [39]. It claims that pigeons are imprinted to the gravity vector at their place of birth, and that this information is stored as a neuronal memory independent of the perception of the actual gravity vector. This would represent an analog to a mechanical gyroscope, which maintains the original inclination of the gravity vector plus the orthogonal horizontal plane after displacement. Thus, at any given point, a gyroscope permits comparison of the angle between a virtual (memorized) and an actual gravity vector converging in the center of the geoid. The comparison of two such vectors with their orthogonal horizontal planes allows for computing azimuth and distance to the point of departure. For a displaced pigeon, this implies that it always senses, under normal gravity conditions, the approximate home direction and distance. It may then find home by either using a map-and-compass strategy with the support of geomagnetic, solar and topographical cues, or it may use a gradient strategy constantly monitoring memorized versus actual gravity vector and reducing the difference. Obviously, such strategies are not mutually exclusive.

In general, the gravity vector theory predicts that pigeons should sense small irregularities of the normally smoothly changing gravity vector. Such irregularities of gravity vector inclinations are found in massive gravity anomalies where they manifest themselves as changes in the horizontal component of the gravity vector. Thus, when pigeons are released from such anomalies, they might deviate from the optimal compass direction for some distance because the birds miscalculate their position in relation to home. During flight, one may also expect occasional directional changes of the flight direction depending on the frequency by which pigeons are assessing the vector differences.

At present, the only approach to experimentally assess the impact of variations in the inclination of gravity vectors on navigation behavior is to study the flight paths of birds near or over strong gravity anomalies. Therefore, during the past four years, we have conducted in the Ukraine a series of studies aimed at elucidating the orientation behavior of pigeons encountering massive gravity anomalies. The Ukraine was chosen because its central part contains massive and well-mapped gravity anomalies distributed in a predominantly flat countryside without any long-distance visual cues. In this paper, we describe a first study with the goal of verifying two predictions made by the gravity vector theory.

In this study, we investigated the orientation behavior of homing pigeons reared within and outside a gravity anomaly and their flight behavior when crossing a gravity anomaly. Thus, we placed one loft in a strong gravity anomaly and another 8 km apart, in a gravitationally normal area as judged by low-resolution gravity maps (scale 1∶200'000). We randomly assigned breeding pairs of local origin to one of the lofts and raised the pigeons under identical conditions. When high-resolution gravity maps (scale 1∶10'000) became available, we realized, however, that the loft in the anomaly-free zone had been placed on a very small but strong local anomaly, the gravity gradient running at right angle to the gradient present at the other loft. For the experiment, the offspring birds from the two lofts were released together from an unfamiliar test site 50 km to the north, from where the beelines to both lofts were almost identical, and birds had to cross the Bandurove gravity anomaly for 10 to 15 km. The gravity theory would predict (i) that pigeons reared in lofts in which the gravity gradients would coincide with the home direction would be better oriented than those whose home loft had a gravity gradient perpendicular to the gravity gradient pointing homewards, and (ii) that pigeons crossing the gravity anomaly should show changes in their direction during flight.

## Materials and Methods

### Pigeons and loft situation

**Figure 1:**
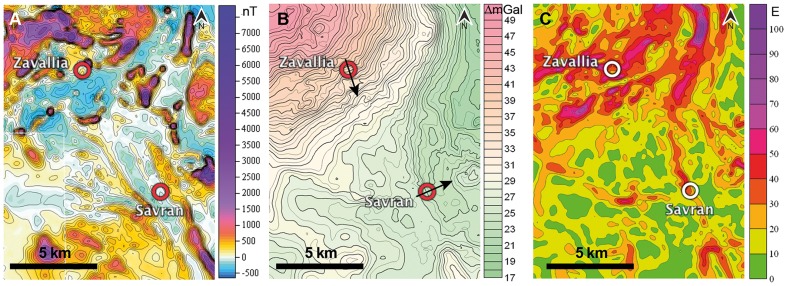
Magnetic and gravity anomalies around the pigeon lofts. The location of the pigeon lofts, Zavallia and Savran, are indicated with a circle. (A) Magnetic anomaly map (nT = nanoTesla). (B) Gravimetric anomalies, the change of the gravity intensity (ΔG_B_ – Bouguer, mGal = milligal). Arrows show the direction of the gravity gradients. (C) Horizontal gravity gradients (E = Eötvös), highest values mark locations with steepest gradient of gravimetric values in border zones of gravimetric anomalies. Note the location of the Savran loft (S-pigeons) on a small yet steep gravity gradient in east-west direction characterized by elevated E-values. For a photographic map illustrating the topography of the area see https://www.dropbox.com/sh/2yrhdtcyzt5uu99/ZFJeNJb0lk.

Two Swiss military pigeon lofts were transferred from Switzerland to Ukraine. One was placed in a village called Savran (N 48°8′, E 30°4′), in a near-normal gravity field (Fig. 1) as evidenced by survey maps. These pigeons are referred to as S-pigeons (Savran-pigeons). After having obtained high-precision geophysical maps, however, we noticed that this loft has been placed on a locally small yet strong irregularity of the horizontal component of the gravity vector (30–40 E), the gradient aligned in a west to east direction (Fig. 1C). For geophysical definitions, see paragraph ‘Topographical and geophysical maps’ below. The other loft was placed in Zavallia (N 48°11′, E 30°0′), only 8 km north in a gravity anomaly, that differed, on average, by 30 mGal from the normal zone (Fig. 1B). These pigeons are referred to as Z-pigeons (Zavallia-pigeons). The horizontal gradient was aligned approximately in a north-south direction, and was of equal strength as the one at Savran (30–40 E), even though the gravity values were much stronger in Zavallia. The Z-loft (Zavallia-loft) was located 1 km from a hill formed by material from a graphite mine of 100 m altitude, and which was visible for humans from a radius of approximately 10 km. We bought 60 pigeons from pigeon breeders from a different region and assigned randomly half to each of the lofts. As soon as the fledglings were ready to fly, they were trained in all cardinal directions around the loft up to 15 km. Early in the training phase, we mounted PVC dummies on the pigeons' back to accustom them to the weight and the size of a GPS logger. The PVC dummies stayed on the pigeons for the whole training period. The GPS loggers were from Technosmart (version GiPSy2) and recorded the position of a pigeon every second with an average accuracy of 4.2 m (in 95% of fixes). The last training release was recorded with GPS loggers. For the Z-pigeons, the training release site was 15 km northeast of their home loft. For the S-pigeons, the training release site was 15 km west of their home loft.

### Experimental releases

**Figure 2:**
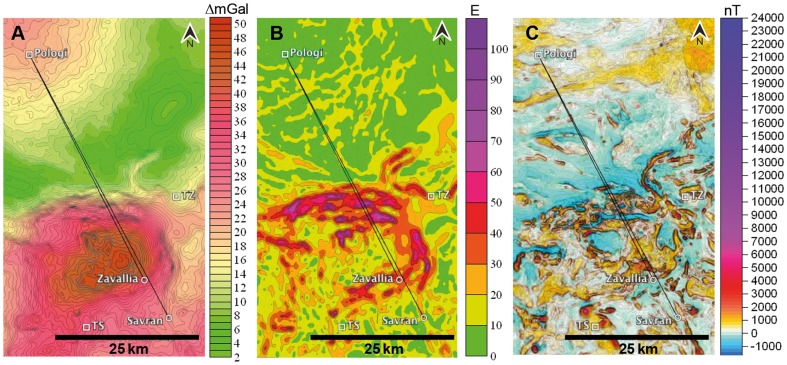
Gravity and magnetic anomalies of the test region crossed by the pigeons. (A) Gravimetric anomalies, the change of the gravity intensity (ΔG_B_ – Bouguer, mGal = milligal); (B) Horizontal gravity gradients (E = Eötvös), highest values mark locations with steepest gradient of gravimetric values in border zones of gravimetric anomalies. (C) Magnetic anomaly map (nT). The square symbols indicate the release sites: Pologi, the experimental release site; TZ, the last training flight to Zavallia loft; and TS, the last training flight to Savran loft. Black lines indicate beelines from the experimental release site to the Z- loft (46 km) and the S-loft (54 km).

The experimental release site Pologi (N 48°34′, E 29°43′) was chosen on the basis of having the same homeward direction for both lofts: the Z-loft, 46 km apart and the S-loft, 54 km apart, outside the Bandurove anomaly (Fig. 2). Since the theory expects that pigeons should derive positional information from the angular difference in gravity vectors between release site and loft, the Z-birds should not experience conflicts with their home gradients (even when these are anomalous) as long as the gradient coincides with the home direction. On the other hand, an imprinted (distorted) orientation of the gravity vector at the home loft might cause a conflict at a release site if it diverges from the home gradient. Before the experiment, we transported the pigeons by car at night to the release site and let them rest a minimum of 4 h until sunrise. Then, we mounted the GPS loggers onto the pigeons' back and released them individually in alternating order, a pigeon from the Z-loft and then a pigeon from the S-loft. We released in total 12 Z-pigeons and 14 S-pigeons on three consecutive days in August 2010 to compensate for possible meteorological variations, and because the number of GPS was not enough to use on all pigeons within one day. We kept 5-minute intervals between releases to prevent pigeons from following each other. After the return of the pigeons to their home lofts, we collected the GPS loggers and downloaded the data to the computer with GiPSy2 software (Technosmart). The weather on the release days on August 26 and 27 was nice with no clouds and no wind. On August 28, there was a southern wind with 10 km/h and again a cloudless sky.

### Analyses

Vanishing bearings, vanishing time and flight track parameters were calculated using the freeware program Wintrack [42]. From the GPS-tracks we determined vanishing bearings and vanishing time (vt) at a distance of 2 km and 5 km from the release site which is in accordance with previous literature [43]. Statistical tests for group differences were performed using the program SPSS and the freeware R. The non-parametric Mann-Whitney-U-test was used to show a difference between the two groups of pigeons in flight parameters and the t-test was used to compare the dispersal distances (dd) of the two groups. Parameters of circular distribution such as the mean vanishing vector (r) [44] were computed with the software program Oriana (Kovach Computing Services). The mean vanishing vector's projection onto the axis of the homeward direction gives the homeward component (hc) showing how homeward orientated the whole group of pigeons was. Circular statistical tests included the Rayleigh-test to show whether the vanishing bearings had a uniform distribution. The Watson-U2-test was performed to reveal any difference between the groups and the Watson-Williams-F-test to depict a difference in the mean vanishing bearings. To quantify the orientation of the pigeons after having left the release site, we measured the dispersal behavior of the pigeons from the beeline from the release site to the home loft (beeline R-H) in steps of 5 km up to 30 km. To this end, circles with corresponding radii were plotted around the release site, and we determined the dispersal distance (dd) from the intersection beeline-circle to the intersection flight track-circle. The dispersal distance has positive values when being east of the beeline R-H and negative values when being west of the beeline R-H (the beeline R-H points from north to south). The maximum distance point was 30 km because 6 of the tracks of the S-pigeons were incomplete and no values were recorded by the GPS loggers for further distances. The analysis of flight tracks parameters aimed to show differences in the general flight behavior between S- and Z-pigeons. The following parameters were calculated from the flight tracks: homing performance (hp), path efficiency (path ef), homing efficiency (hom ef), path linearity (path lin) and GPS speed. Homing performance was calculated by dividing the beeline distance R-H by the duration of the flight (km/h) and is an indicator of how fast and straightforward the pigeon flew to its home loft. Path efficiency is a similar measure but takes into account the whole track length instead of the time; it is the beeline distance between the release site and the home loft divided by the track length in %. Homing efficiency adds the homeward component as percentage of the track with a homeward component >75%. Path linearity is the sum of the ratio of the distance between two positions 32 s apart and the track length of two positions 32 s apart in % and shows how straight the pigeon's tracks were regardless of the home direction. The GPS speed is the ground speed in km/h excluding rests.

A second analysis was conducted to compare the flight behavior of the pigeons just before the anomaly, when crossing the border zone of the anomaly with a steep change in the horizontal gradient of gravity and when flying within the anomaly. The same flight tracks of the experimental release were used, but only of the Z-pigeons because most of the S-pigeons did not cross the anomaly. Three zones were defined with a width of 3 km each: zone 1 corresponding to the non-anomalous area in front of the anomaly, zone 2 corresponding to the border zone of the anomaly and zone 3 corresponding to the core anomaly area. Flight parameters such as the flight duration, path efficiency (path ef), path linearity (path lin) and GPS speed were calculated for each part of the pigeon's flight track within the three zones. First, a non-parametric repeated measures analysis of variance by ranks, the Friedman-test, was used for each parameter. If the test showed a significant difference in the means of a parameter, we then used the Wilcoxon signed-rank test to compare the flight parameter in the different zones.

Of the 12 released Z-pigeons, 12 vanishing bearings and 11 flight tracks could be used for analysis. One GPS track was excluded because it did not record the full flight path. Of the 14 released S-pigeons, 11 vanishing bearings and 4 flight tracks could be used for analysis. Three S-pigeons were lost and 6 were late returners (>5 h) of which none had a fully recorded flight path. One flight track was excluded but only in the flight parameter analysis (n = 4) because it was an outlier, overshooting the home loft and continuing a long journey south of the home loft.

### Topographical and geophysical maps

Flight tracks were visualized with the aid of the freeware program QantumGIS. Geophysical maps present Bouguer gravity anomalies obtained by gravimetric terrestrial surveying. Bouguer anomalies are typically corrected for latitude, topographical elevation above sea level and soil thickness, and are expressed in ΔGal (indicated as ΔmGal in Figures and simply as mGal in the text). The modulus of horizontal gravity gradients was calculated by using the Bouguer anomaly data: gravity difference in neighboring points, divided by the distance between these points. The gradient is usually measured in units of Eötvös (E). One E corresponds to 0.1 mGal/km. There is thus a strong correlation between Bouguer maps and horizontal gradient maps: high values of E occur in the border zones of strong gravity anomalies (Fig. 1 and 2). Figuratively, these zones indicate regions wherein the vertical direction of a plumb is slightly tilted by a laterally situated underground inclusion or lack thereof, whereas in the center of a gravity anomaly, the direction of a plumb coincides with the theoretically expected direction to the center of the earth.

Gravity maps include different levels of resolution. For example that in Figure 1 is largely based on a grid of 100×100 m with an accuracy of 0.1 mGal. The other maps were composed from terrestrial surveys including cell grids of 250×250 m, 250×500 m, and 500×500 m. Magnetic maps were composed from aerial (50 m altitude) and terrestrial surveys (observation lines of 100 or 250 m distance, respectively). The contour interval on the maps of the magnetic field is 50 nT.

The frame of the gravity gradient map shown in the Figures in yellow color-coding denotes the changes in the horizontal gravity gradient from 0 to 50 E with an abrupt variation on the northern border of the anomaly. The average Bandurove gravimetric amplitude, which is the difference between the value at the center of the anomaly and the mean anomaly in the environmental field, is 30 mGal. The amplitude from different sides of the anomaly is 40 mGal from the north, 35 mGal from the west, 28 mGal from the east and 20-13 mGal from the south. The release site Pologi shows a magnetic intensity of 329 nT, the homeloft area in the village Zavallia 384 nT and the homeloft area in the village Savran 206 nT (Fig. 1A).

### Ethics Statement

The experiments were conducted according to Swiss regulations on animal welfare and experimentation, licenses 99/2008 and 92/2011 issued by the Zurich Cantonal Veterinary Office. The above government licenses are only issued after having been approved by an ethics committee including scientists and animal protection organizations. The approval is not shown to the applicants (who apply directly to the government). Keeping homing pigeons and conducting pigeon releases in the Ukraine does not need governmental permission. Homing pigeons are not an endangered or protected species. Pigeon racing is a popular sport as in many other countries worldwide, including the US, all European and many Asia countries. The lofts were placed on private grounds on a rental basis with the permission of the landlords.

## Results

### Comparison of pigeon groups

All Z-pigeons (n = 12) arrived at the home loft and were continuously homeward oriented. In contrast, S-pigeons (n = 14) were not homeward oriented and showed poor homing performance: we lost 3 pigeons and 6 were late returners (>5 h).

**Figure 3:**
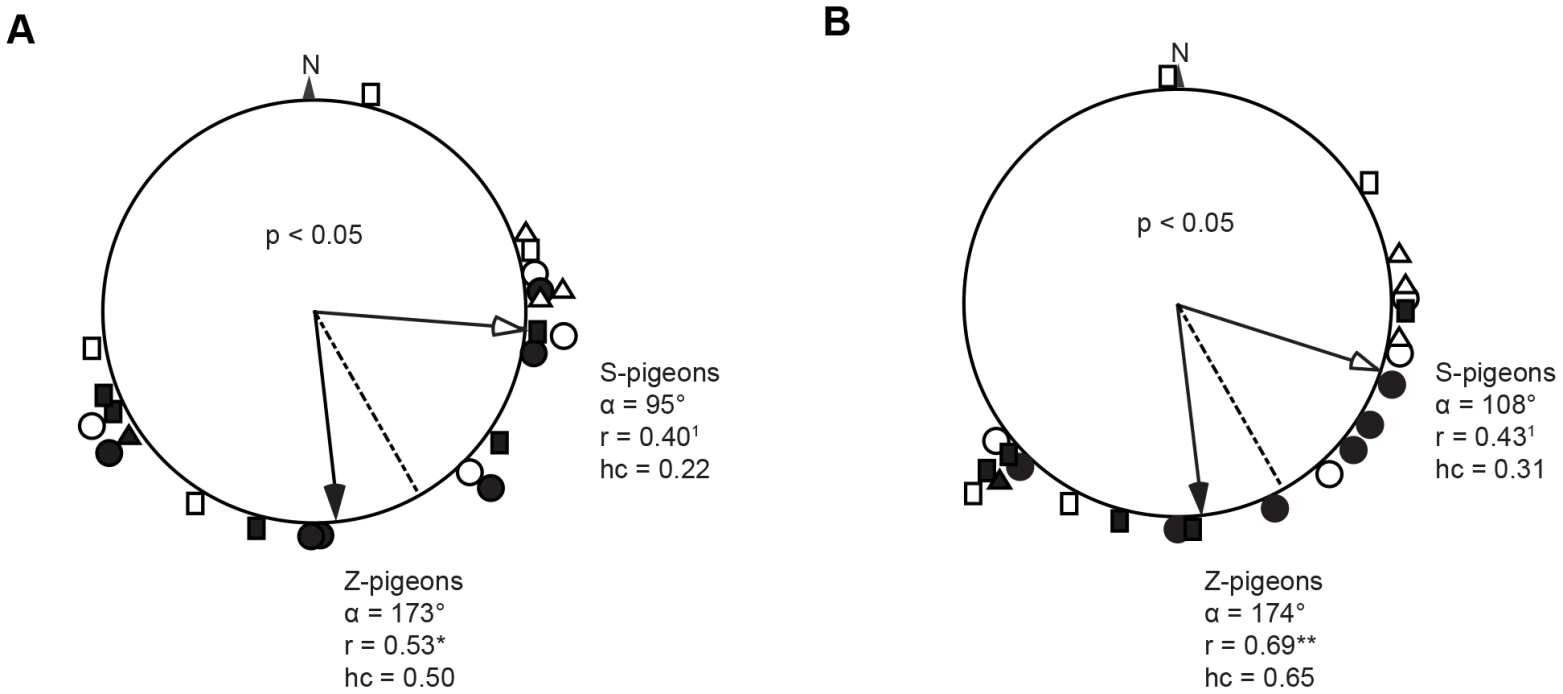
Vanishing bearings of Z- and S-pigeons at 2 and 5 km. (A) Vanishing bearings of Z- and S-pigeons at 2 km and (B) at 5 km from the release site. The black symbols refer to Z-pigeons (n = 12) whereas the white symbols refer to S-pigeons (n = 11). Circles, rectangles and triangles indicate pigeons released on August 26, 27 and 28, respectively. The bold arrows show the mean vanishing bearings of the Z-pigeons with a black arrow head and of the S-pigeons with a white arrow head. The dotted line shows the home loft direction, 152°. α is the mean vanishing bearing, r is the mean vanishing vector and hc is the homeward component. The difference between vanishing bearings of the Z- and the S-pigeons was calculated with the Watson-Williams-F-Test for significance (p-values in the circular diagrams). The significance levels for the Rayleigh test (r) are indicated with ^1^ = not significant, * = p<0.05, ** = p<0.01.

The S-pigeons showed a significant poorer initial orientation compared to the Z-pigeons and the vanishing bearings of the Z- and the S-pigeons were significantly different from each other (Fig. 3). The distribution of the vanishing bearings of the Z-pigeons was significantly different from random (parameter r, Fig. 3A), whereas the vanishing bearings of the S-pigeons showed a random distribution (parameter r, Fig. 3A). The mean vanishing bearing of the S-pigeons deviated from the home direction by 57° ± SD 77°. Many S-pigeons headed first north and northeast. The S-pigeons spent also more than double the time flying (vt) around the release site within 2 km distance than the Z-pigeons but the difference is not significant (S-pigeons vt mean: 11.1 ± SD 12.4 min, Z-pigeons vt mean: 4.7 ± SD 4.3 min).

The poor initial orientation of the S-pigeons did not change when examining their vanishing bearings at 5 km distance from the release site. At 5 km, the vanishing bearings were still not different from random (parameter r, Fig. 3B). The mean vanishing angle still deviated from the home direction by 44° ± SD 75°. In contrast, the Z-pigeons were better oriented at 5 km compared with the results at 2 km with a higher homeward component and a stronger mean vanishing vector (parameter r and hc, Fig. 3B). The difference between the mean vanishing vectors of the two groups was also significantly different at 5 km distance from the release site.

**Figure 4:**
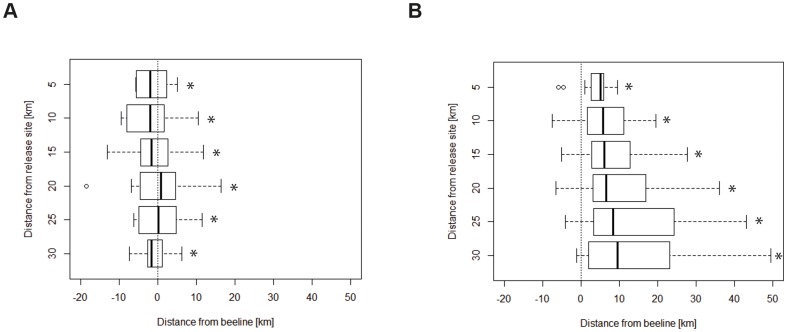
Pigeons from the two lofts maintain different flight directions. (A) Distances of the Z-pigeons from the beeline at 5 km steps. (B) Distances of the S-pigeons from the beeline at 5 km steps. Points easterly of the beeline R-H correspond to positive values of the x-axis, whereas points westerly of the beeline R-H correspond to negative values. The box ranges show the upper and lower quartile with the median, and whiskers extend to the most extreme data point no more than 1,5× the interquartile range. Points outside the range are outliers. The stars indicate significant differences between the Z- and the S-pigeons (t-test, p<0.05 for all).

The pigeons not only differed in their initial orientation but deviated continuously from the homeward direction at distances up to 30 km from the release site (Fig. 4). The positions of the S- and Z-pigeons at 5, 10, 15, 20, 25 and 30 km distance from the release site were always significantly different from each other (t-test, p<0.05 for all). The number of Z-pigeons is always 11 for all distances; for S-pigeons, it is 11 up to 15 km, at 25 km there were only 10 and at 30 km 9 birds. The S-pigeons showed a strong bias towards the east: the median of all distances lies always eastern of the beeline R-H (positive values in Fig. 4B) and the scatter of the data increased with the distance from the release site. The Z-pigeons scattered the most at 15 to 20 km distance from the release site but then converged again when approaching the home loft (Fig. 4A).

**Figure 5:**
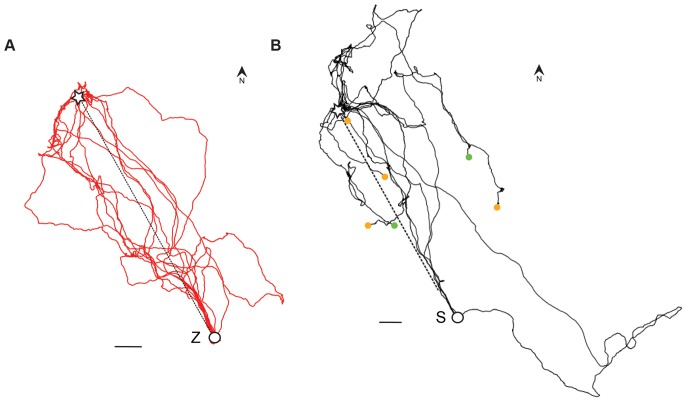
Flight tracks from the same experimental release site. The dotted line is the beeline from the release site, indicated with a star, to the home lofts. (A) The flight tracks of 12 Z-pigeons flying to their home loft Z (Zavallia). The distance from the release site, to the Z-loft is 46 km. (B) The flight tracks of 11 S-pigeons flying to their S-loft (Savran). The distance from the release site to the S-loft is 54 km. An orange dot indicates that the pigeon stopped flying and was pausing until the GPS ran out of battery power. A green dot indicates that the pigeon was still flying while the GPS ran out of battery power. The bars in both pictures represents 5 km.

Figure 5A depicts the flight tracks of 12 Z-pigeons (but only 11 tracks were used for analysis): the homing performance was 51 km/h and they flew with an average speed of 69 km/h. The path efficiency was 72% and an average of 71% of the track was homeward oriented (homing efficiency) with a path linearity of 94%. In Figure 5B, 11 flight tracks of S-pigeons are illustrated, but 6 flight tracks are incomplete and stop (indicated with orange and green points). In total, we calculated flight track parameters of 4 S-pigeons that returned home: they did not differ significantly from the Z-pigeons in homing performance (40 km/h), homing efficiency (58%), path efficiency (62%) and GPS speed (68 km/h, Mann-Whitney-U-test). However, the S-pigeons flew significantly more tortuous than the Z-pigeons with a path linearity of 90% (p<0.05, Mann-Whitney-U-test).

### Analysis of tracks within the Bandurove anomaly

**Figure 6:**
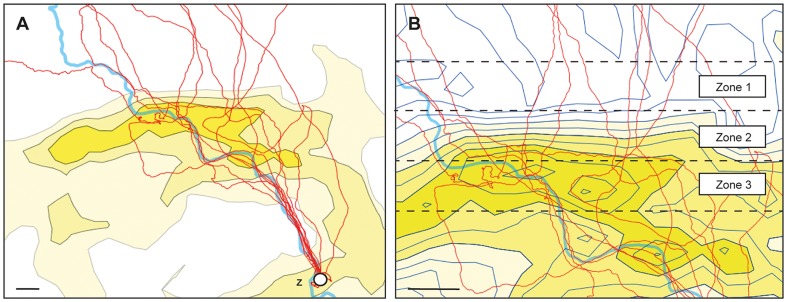
Flight tracks of Z-pigeons crossing the Bandurove anomaly. (A) Flight tracks of 11 Z-pigeons. Z depicts the home loft. The thick meandering blue line is the Bug river. The contour lines of the gravity anomaly (horizontal gradient) are in steps of 10 E. The brightness of the color denotes the anomaly intensity: light (E = 20), middle (E = 30), dark (E = 40). 1 E = 0.1 mGal/km. (B) Close-up of the same map but with blue contour lines in steps of 5 E. Zone 1: normal gravity area before the Bandurove anomaly; zone 2: gravity anomaly area with a steep change of the horizontal gravity gradient (isolines are close together); zone 3: the core anomaly area with continous values of 40–50 E. The bar in the lower left corner of both pictures represents 3 km.

The flight tracks of the Z-pigeons superimposed on a scheme of a horizontal gravity gradient map are depicted in Figure 6. The comparison of the flight behavior of the Z-pigeons before crossing the border of the anomaly and flying within the anomaly revealed following results: the comparison of the means of each flight parameter (path efficiency, path linearity, GPS speed and time) only showed a significant difference of the parameter path linearity between the zones (Friedman-test, p<0.05). The Wilcoxon signed-rank test revealed that significant differences could be attributed to the comparison of the parameter between zone 2 and zone 3, and zone 1 and zone 3 (p<0.05 for both). There was no difference between zone 1 and zone 2, i.e. the flight tracks became more tortuous only within the core anomaly (zone 1 path lin = 98% ± SD 1.7%, zone 2 path lin = 98% ± SD 1.4%, zone 3 path lin = 97% ± SD 2.5%). All other parameters were not significantly different when tested with the Friedman-test. Path efficiency was in zone 1 83% (± SD 11.3%), in zone 2 78% (± SD 15.0%), and in zone 3 66% (± SD 31.2%). The GPS speed was in zone 1 66 km/h (± SD 12.3 km/h), in zone 2 65 km/h (± SD 11.5 km/h), and in zone 3 66 km/h (± SD 12.2 km/h). The flight time was in zone 1 3.4 min (± SD 0.9 min), in zone 2 4.2 min (± SD 2.6 min), and in zone 3 5.9 min (± SD 3.9 min).

### Inspection of individual flight tracks within the gravitational anomalies

When investigating in detail the individual flight tracks of the Z-pigeons, most of them showed more tortuous flight paths within the core of the Bandurove anomaly area at 18 km northwest of the home loft (Fig. 6). One individual bird, after crossing the border zone of the anomaly, abruptly changed its southerly flight course to the west for 4 km, then to the south for 4 km, just to turn to the east, shaping a square with its flight course. Another example of a bird changing its flight course within the anomaly was a pigeon flying southwest, then changed abruptly to southeast, but then, after 5 km, changed to fly northeast for 5 km, circled and then flew south, homewards. Two birds started following the river already before the anomaly, one of them suddenly flying four small circles within the core anomaly, the other following the river closely until it reached the home loft. Nine out of 11 pigeons aligned to the river 8 km north of the home loft that led them directly home.

**Figure 7:**
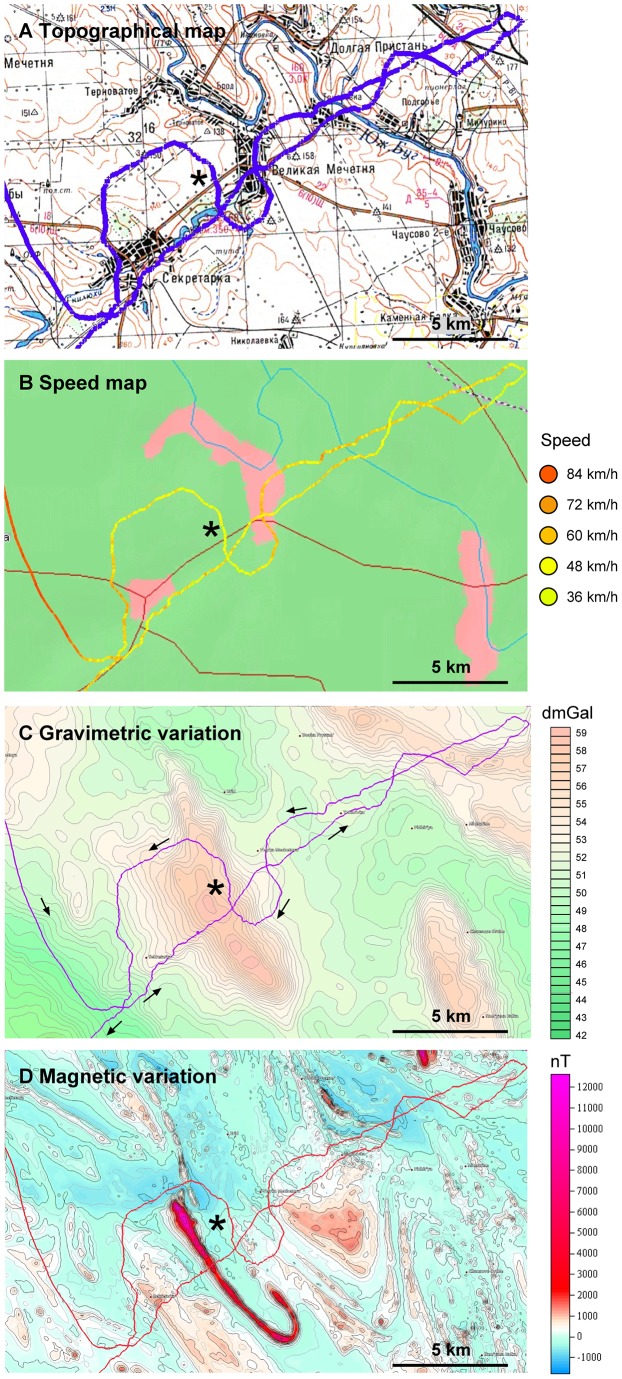
Flight path of a pigeon crossing gravity and magnetic anomalies. (A) Topographical map of Sekretarka region. (B) Map showing flight speed of pigeon 305. Note the sudden reduction in speed when approaching the anomaly; reduced flight speed is then maintained throughout the region. (C) Gravimetric anomalies. Densely spaced isolines indicate those regions with irregularities of the horizontal gravity gradient. (D) Strong magnetic peak on top of the gravitational anomaly. Asterisk denotes the position of a former Sovjet SS-18 rocket launch station.

Among the S-pigeons, one bird showed a peculiar behavior (outlier in Fig. 5B). The pigeon departed rapidly with high speed (>80 km/h) in easterly direction, changed the course after 5 km in southeasterly direction (139°) and continued straightforwardly with high speed for 75 km, thus missing the home loft. It then suddenly reduced flight speed to 40–50 km/h and turned in a right angle towards northeast, into a region containing numerous gravity anomalies (the Sekretarka region, Fig. 7B). It then adopted a tortuous flight course passing in-between two gravity anomaly peaks and maintained that course until it hit another gravity anomaly where it turned 180°, flying back in the direction it came from. Upon approaching the anomaly region passed before, the bird changed the course apparently aligning to the contours of the gravity anomaly (Fig. 7C), thereby flying around the anomaly. Exactly within the anomaly was a former missile station for intercontinental rockets probably placed there because of the anomalous geophysical values. Afterwards, it made again a sharp turn and flew 40 km NW to reach the home loft. The Sekretarka region contained also a localized magnetic anomaly peaking up to 10’000 nT (Fig. 7D). Yet the flight track aligned much better with the border zones of the gravimetric anomaly.

### Inspection of training flights

Given the unexpectedly poor performance of the S-pigeons, we analyzed carefully the flight paths of both Z- and S-pigeons during their last training flights in order to check for a directional bias at the experimental release site.

**Figure 8:**
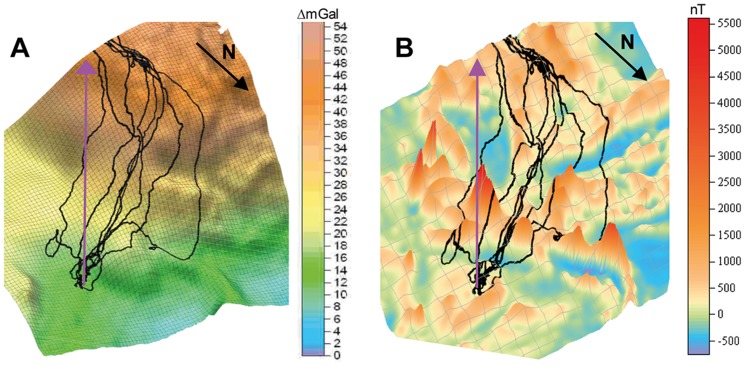
Last training release of Z-pigeons northeast of the Zavallia loft. (A) Flight tracks on gravity anomaly map. Most birds appear to follow the steepest gravity gradient of the anomaly, then turn left when they hit the Bug river. Scale 0–54 ΔmGal. (B) Flight tracks on magnetic anomaly map, showing partial coincidence of magnetic and gravity anomalies. Scale -500 to 5'500 nT. Violet arrow shows home direction. For a topographical map: https://www.dropbox.com/sh/2yrhdtcyzt5uu99/ZFJeNJb0lk.

The last training release of the Z-birds occurred 15 km NE of the Z-loft under conditions when they could have easily seen the artificial hill marking the position of the home loft by taking the beeline direction of 200° (Fig. 8). Instead, they all deviated westerly from the beeline, following initially a flight path (225°) along the steepest gravimetric gradient (coincident with high E values, Fig. 8A). Approximately at the level of the same Bouguer levels as their home loft, they began to turn southward, eventually following the Bug river for another 5–7 km to their home loft. Flight tracks coincide much less with the 3D-topography of the magnetic anomaly since the pigeons crossed several magnetic peaks close to the release site (Fig. 8B). Looking at the initial vanishing behavior of the experimental release site Pologi, we found 4 tracks for which a training bias might account for (mean vanishing vector at the training site: 224°).

**Figure 9:**
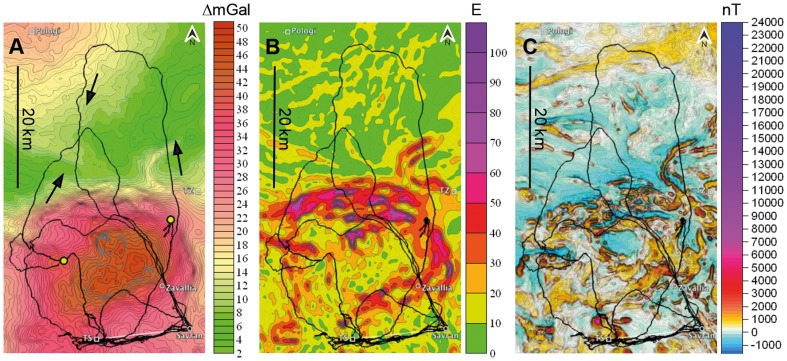
Last training release of S-pigeons west of the Savran loft. (A) Flight tracks on map of gravimetric anomaly, i.e. the change in gravity intensity. Yellow dots indicate birds resting more than 5 h. Three pigeons made long journeys to the north into the anomaly and back. Arrows show flight direction. (B) Tracks on a map showing irregularities of the horizontal gravity gradient (corresponding to the steepest gradients of gravimetric values). (C) Same tracks on a map with geomagnetic anomalies. The overall geomagnetic variation is relatively low (between -1000 and 1000 nT), with scattered peaks of higher intensity. For a topographical map: https://www.dropbox.com/sh/2yrhdtcyzt5uu99/ZFJeNJb0lk.

On the other hand, the tracks of the training flights of the S-pigeons revealed a much more variable pattern (Fig. 9). The initial vanishing orientation was random with a mean vanishing vector pointing north (348°). Three birds flew first in western direction for 4–5 km, then turned and flew directly home. Five pigeons headed northward towards the Bandurove anomaly, of which only one pigeon corrected the flight course homewards. Four S-pigeons, however, showed long journeys from 40 to 120 km within or even beyond the Bandurove anomaly. These four pigeons differed in their flight behavior at the experimental release because only one of them flew home in a direct course. Due to widely differing initial orientation during the training flight, there was clearly no directional bias at the release site.

## Discussion

Pigeons reared in lofts located on gravity anomalies with diverging horizontal gravity gradients showed, as theoretically anticipated, a significant difference in their vanishing bearings from the same release site in a normal gravity area. The Z-pigeons were significantly homewards oriented while the S-pigeons showed random orientation. Furthermore, the S-pigeons were not only initially disoriented, but also showed prolonged disorientation up to 30 km from the release site and some pigeons never found back home. The few S-pigeons that did home did not differ in their flight behavior to the Z-pigeons that homed all successfully. The Z-pigeons that crossed the anomaly did change their flight behavior within the anomaly in comparison of a 3 km zone in front of the anomaly. Within the core gravity anomaly, they showed more tortuous paths. Thus, both a specific (initial orientation) and a general prediction (reactions to anomalies) of the gravity vector theory were fulfilled. However, we realize that the results might be subject to different interpretations. We will thus discuss first the problems of initial orientation, then reactions to gravity anomalies and, finally, the relation of gravity and geomagnetic anomalies.

### Initial orientation

The initial orientation of pigeons is subject to release site specificities, training effects, and home loft conditions. The release site was in an open field in a flat topography, the next village 1.5 km to the northeast. The distribution of the vanishing bearings of the S-pigeons was random, but 6 pigeons flew eastward, in the direction of the gradient characterizing the position of the home loft. One possible explanation is the influence of directional training [45]. However, the pigeons had not been trained in one specific direction but in all cardinal directions. Therefore only the last training release could have had an effect on their vanishing behavior. Yet, as shown in the description of the training flights, the initial orientation at the training site was very scattered and only two S-birds flew both at the training and the experimental release site to the east. As for the Z-pigeons, the mean vanishing vector was close to the homeward direction and only 3 pigeons vanished in the previous training direction, therefore also diminishing the effect of training on initial orientation. The third and possibly most important explanation for vanishing bearings of homing pigeons is the location and the condition of the home loft. Our two lofts were exactly identical, two former Swiss army lofts, populated with comparable numbers of pigeons, both placed in a garden with an outlook within a village and fed the same diet. Both pigeon groups had similar training experience and were trained always by the same person. Thus, it appears unlikely that this type of loft-specific factors affected the results.

However, studies have shown that pigeons from a given loft have a consistent directional bias at different release sites [46]–[47], [43]. For example, pigeons from neighboring lofts showed divergent vanishing bearings at the same release site [47]–[49]. The latter study is of relevance to our data as it compared the vanishing bearings of sibling pigeons raised outside and within a magnetic anomaly, and being released at various magnetic anomalies. The birds raised in the anomaly were significantly disoriented at one site but not at other sites. Walcott speculated that the birds from the two lofts had developed different map-forming strategies in accordance with their early experience with geophysical parameters. This seems to be the most likely explanation for our data. The surprisingly poor initial orientation and the tracks observed during the training flight from 15 km suggest that several S-birds had experienced a problem in establishing a navigational map, while the Z-birds were mostly well oriented during both training and experimental release. This conclusion is supported by the observation that the flight tracks of the S-pigeons continued to be misdirected up to 30 km. It might be a coincidence that the different vanishing bearings reflected the diverging horizontal components of the gravity vector at the home lofts, but one cannot rule out that the small local gravity anomaly (horizontal gradient) at the S-loft with gradients perpendicular to those of the Z-loft was contributing to the orientation problem of the S-birds. However, if true, this would imply that extremely subtle differences in orientation of the horizontal gradient of gravity might produce a loft-specific bias in flight bearings at release sites. Clearly, this observation must be verified by releasing pigeons from lofts with normal gravity conditions at places located in the border zone of gravity anomalies biasing the initial orientation of the birds.

### Crossing the gravity anomaly

The flight course of pigeons is affected by many factors, however, their effect also depends on which navigational strategy the bird applies [50]–[51]. Inspection of individual tracks is necessary to interpret the results in a meaningful way. In our experience, gathered during the last 10 years, one individual flight strategy is called “compass flight”, in which a pigeons flies in a straight line neither paying attention to distracting topographic stimuli as villages and rivers, nor to geophysical anomalies. In our data, some pigeons adopt a straight directional flight at a high flight speed between 70–80 km/h shortly after the release site. Another flight strategy is when pigeons maintain a slower search-type flight pattern and are either guided or distracted by external factors such as landscape features, other pigeons and possibly also geophysical cues. This flight pattern is often associated with prolonged rests. Such birds are more likely to follow gravity gradients or magnetic isolines but often not exclusively. Some of our flight tracks suggest that the birds sense gravity anomalies during flight, although they do not appear constantly guided by gravity variations. The analysis of the flight tracks of the Z-pigeons approaching the Bandurove anomaly from the north showed 3 compass flyers crossing the anomaly at high speed, yet even these showed a minor flight correction in the border zone of the anomaly. The other more slowly approaching birds did not immediately react when crossing the steep horizontal gradient of the gravity vector but about 3 minutes later, when flying within the core anomaly: they showed more tortuous flight paths and sometimes abrupt changes in their flight course, e.g. one pigeon turned from flying south to flying northeast and another pigeon altered its southerly course to heading to the west, to the south and back to the east, all within the anomaly. Probably the sudden change of the perceived gravity vector when crossing the anomaly from the north irritated the pigeons and led to a search behavior indicated by greater path tortuosity. Two pigeons started following the river already within the core anomaly, possibly perceiving familiar olfactory cues from the river. Other pigeons followed the river later, after the anomaly, 7 km in front of the home loft. The last training release for the Z-pigeons also showed that approaching the Bandurove anomaly from the east itself did not cause problems. Instead of taking the direct route to the loft along the beeline, the pigeons followed the steepest gravity gradient to the southwest and then aligned their flight course to the river. Normally, flight tracks over flat countryside scatter equally to the left and right of the beeline, but there was only one bird flying initially to the left of the beeline and even this one corrected the flight path to the right side of the beeline.

There were two other examples suggestive for sensing gravity anomalies. The first observation was the behavior of the S-birds during their last training flight. Almost half of these pigeons (5 out of 13) appeared to be attracted by the northerly lying anomaly, notably devoid of any distinct topographic features. In case of a non-systematic problem of initial orientation, one would have expected that at least some birds would be heading also southward, but none of them did so. The second example was an S-bird that flew first with high speed along a (wrong) compass direction, despite of the fact that he had visited the area two days before. It changed its flight path and flight behavior suddenly after having passed the loft. As shown in Figure 7, the bird appeared to sense a gravito-magnetic anomaly easterly of its flight path. Notably, this anomaly appears barely on large-scale maps but on high-precision maps, the gradients from the bird's position to the anomaly were as high as the Bandurove anomaly (about 40 E difference), associated with a local geomagnetic anomaly peaking at 10’000 nT. Possibly, the gradients were reminiscent of the familiar Bandurove anomaly near the birds' homeloft. On its way back, the pigeon circumvented the gravity anomaly rather precisely along the gravity isolines, before eventually turning home. Since it followed the contours of the gravity anomaly about 1.5 km before the sudden peaking of the magnetic anomaly, on might at least tentatively conclude that this location possessed some highly distinct geophysical properties. That the pigeon also circumvented a former intercontinental missile silo might be coincidence, but given the reliance of cruise missiles and adversary rockets on gyroscopic (i.e., gravity vector) information, that place was at least well chosen by the constructors.

### Gravity and geomagnetic anomalies

Gravity anomalies caused by underground densities containing magnetite frequently overlap with magnetic anomalies. For example, geomagnetic anomalies have been reported to influence the flight paths of GPS-tracked pigeons [32] in a manner similar to what has been partially observed by us, namely having a preference of aligning or crossing at right angles strong anomaly gradients. Interestingly, in Dennis's study, the correlation between geomagnetic and gravity anomalies was significant (r = 0.62).

In order to avoid the concurrent influence of magnetic and gravity anomalies, Lednor and Walcott [41] investigated the orientation behavior of pigeons flying from the center of gravity anomalies located over salt domes with less density and therefore producing a negative gravity anomaly with little magnetic variation. The amplitudes of the anomalies ranged from -2 to -10 mGal, suggesting that gravity differences in this order have less or no impact. In comparison to the salt domes, the Bandurove gravity anomaly is positive and much stronger with ranges from 20 to 40 mGal. Perhaps more importantly, this anomaly is also more massive, caused by a tectonic break with locally interspersed magnetic and gravity anomaly peaks. One should also note that the gravity vector theory does not predict altered vanishing bearings from the center of anomalies but expects deviations from the home direction primarily for releases from border zones of anomalies.

In agreement with our observations, Dornfeldt [38] compared weaker (-9 to 14 mGal) gravity anomalies to stronger gravity anomalies (15 to 49 mGal) and found that pigeons were significantly less homeward oriented and homed slower from the stronger gravity anomalies. Supported by an extensive and detailed multivariate analysis including geomagnetic variation, topography and weather conditions, he concluded that gravity parameters form an essential part of the pigeon's map sense.

In our study, there were a few small magnetic anomalies around both home lofts but not much variation at the release site Pologi and within the Bandurove gravity anomaly, e.g. the intensity difference from the release site 2 km in the home loft direction is as little as -16 nT. This value is generally considered as geomagnetic noise. Thus, it appears unlikely that the differences in initial orientation of Z- and S-pigeons were caused by geomagnetic variations. An apparent fact is that compass and position finding mechanisms based on the inclination angle of the earth's magnetic field are calibrated against the gravity vector. Thus, an irregularity of the gravity gradient may entail a wrong reading of the magnetic inclination angle. There are several arguments against such an interpretation: (i) the magnetic inclination angles provide only information on latitude (the so-called longitude problem). Thus, should the pigeons indeed use magnetic inclination angles for orientation, one would expect less navigational problems caused by gravity anomalies with a north-to-south gravity gradient, because magnetic and gravitational cues coincide. This has been observed for the Z-birds. On the other hand, a west-to-east gravity gradient at the pigeon's birth place should not bias the north-to-south inclination angle of the geomagnetic field in a geomagnetically normal region, but if the pigeon perceives both cues, it is likely to cause conflicting information at the release site, leading to a dispersal of flight paths such as observed in the S-pigeons. (ii) Phylogenetically, a gravity-based navigation system would appear to be much more stable and preferable for migratory species and long-distance navigators that depend critically on precise navigation, because the magnetic field of the earth is constantly fluctuating and shifting its poles. (iii) Pigeons released from sites with geomagnetic anomalies appear to correct their flight paths soon after reaching normal territory [34], while the pigeons released here maintained wrong flight directions over long distances. This might indicate that variations of the horizontal component of the gravity vector appear to influence the map sense of the pigeons, while possible covariations with magnetic parameters appear to have only a short-lasting impact on compass mechanisms.

### Conclusions

1. Our data largely fit the predictions made by the theory formulated by Kanevskyi [39], namely that birds reared in locations with different gravity gradients show different initial orientation and temporary changes in flight tracks when encountering sudden massive changes in gravity gradients.
2. The GPS tracking data from this study confirm Dornfeldt's earlier observations of altered vanishing bearings of pigeons at release sites with gravity anomalies [38], and are in line with the study of Larkin and Keeton on effects of lunar cycles on vanishing bearings [37].
3. It remains likely that at least some of the altered orientation of pigeons at release sites with geomagnetic anomalies reported by other studies may have been caused by gravity anomalies. Thus, it would seem advisable to provide gravity maps systematically for release studies aimed at assessing geophysical and other parameters.
4. Upcoming studies should further investigate whether releases at strong positive or negative gravity anomalies, with and without accompanying geomagnetic anomalies, can confirm the observed problems in orientation of pigeons, specifically misleading cues provided by gravity gradients.
